# The Chemical Treatment of Diseases

**Published:** 1861-09

**Authors:** C. B. Hall


### THE CHEMICAL TREATMENT OF DISEASE.

BY C. B. HALL, M. D.

The short time I can spare for an essay could be readily occupied in naming the different hypothesis advanced for the explanation of the *modus operandi* of medicines, all at variance with one another, all failing when put to the test of practice, and yet none without some grounds of physiological truth for their foundation.

I propose, therefore, that we leave as we find them those sound principles of nosology that have stood already the experience of men of learning and thought, and devote a few minutes to the consideration of Liebig and Muller's opinion that inflammation is an oxydized state of the proteine, and that all disease is the result of disarrangement of the affinities of particles, and see how far a chemical treatment may serve as an adjunct to a regular course of medicine. We do know of strange animal changes constantly attending the animal economy. Thus, in the normal state, the gastric juice, the almost first stage in nutrition, is acidulous, while the blood, the result of this digestion, is alkaline. Again, we have the secretion from the liver, the largest secreting organ in the body, with an alkaline base, while the product of the no less important organs, the kidneys, is uric acid. We have also the oleaginous and albuminous secretions, the representatives of nitrogen and carbon, as we find others of oxygen and hydrogen, the two other elementary principles of all organic compounds. This in the healthy state. How innumerable the effects of their slightest variation in disease! not acknowledging the theory that this constitutes disease, but simply viewing them as coincidents and their regulation as concomitants.

Take, for instance, the simplest form of congestion, or perhaps more properly, torpor of the liver, found in the moderate drinker, particularly the beer-drinker, and more particularly when in moderation he has taken a little extra, with a few glasses of spirit; you find the tongue coated with heavy white fur, the gums pale and the fauces dry, the patient complaining not so much of constipation of the bowels as a difficulty in passing what he calls a gummy, sticky sort of substance, which clings to him with the tenacity almost immovable, and of a dark green color, with very little odor, and attended by smarting, but no pain. The usual remedy for this is blue pill and black draught, or, as an old friend of mine in the country takes, ten grains of submurias hyd., followed by salts and senna. Chemically this is an acidulous excess, both in stomach and liver, and ten grains of carbonate of soda to act on the stomach, and ten of bitart. potass. to neutralize the hepatic secretion, in a glass of cold water, will often effect a cure in a few hours.

One of the most troublesome attendants of bilious as well as of infantile remittent fever, is the constant passing of green bile with mucus, showing its irritating effect on the membrane, thus provoking the febrile action and otherwise retarding the cure. I do not mean to say that liq. potass. or any other preparation of that alkali will cure bilious fever, but there is no doubt their use will correct this abnormal secretion, and thus effect one of the most important indications.

On the treatment of dysentery or diarrhœa, or whatever name you give to the various bowel complaints of children, you find a double action, or one extreme running into the other. If you are consulted in the early stages, you find the tongue slightly coated, but white, appearing as if the child had just taken a drink of milk. The stools green, somewhat painful, but not frequent, &c. This is usually treated with antacids, as eyd. c. creta, with creta c. opii cemp., or barb. soda, so that I have no particular point to call your attention to. But what is far more likely, you do not see the case till various pills and potions have been administered by the too confiding parents, suggested by the too-knowing neighbors, *whose children have been exactly the same*, and cured by the far-famed remedy. You find the tongue coated in the centre with a dirty white, inclining to brown, the tip and sides red, the fauces, gums and lips of the same color; a painful expression of countenance, with a whining, feeble cry, constantly picking the lips or ends of its fingers; stools more frequent, of the color of the coating of the tongue, more painful before each motion, and increasing in frequency, &c., and you will invariably find an alkaline reaction, the stools often effervescing with nitric acid. Whatever course of treatment you would suggest, you would find its efficacy most wonderfully advanced by an acid accompaniment, such as tr. ferri muriatis. Or still further, you might find the eye sunken, with a dark areola; skin something of the color of the tongue; flesh full, but flabby and doughy, with other strumous indications. Here is an opportunity for a double chemical action. Feed the child on starch, and give diluted nitric acid. You will not only furnish the best nourishment, and counteract the excess of alkali in the system, but nitric acid converts the starch into oxalic acid, than which no remedy appears to have more specific power over the strumous diathesis.

Take another familiar example with children, one in which you have no doubt been sorely tried, and wished, like the man of old, "your enemy would write a book" on it. A child at breast—the mother, strong and healthy, eats her meals with relish, has plenty of milk for the child, even more than it requires. This you find, on standing in the glasses, rich, and covered with thick, almost buttery cream. She tells you the child nurses freely, and throws it up without any curdling. Bowels inactive for a few days, then three or four motions a day for a few more. Pulse, feverish, child pale, fretful, crying and whining constantly. Here is a case of infantile indigestion, tending to cachexia. You prescribe infusion of cinchona, or some tonic, but without avail. Chemistry says, if you give that child sugar, it will convert the casein of the milk into lactic acid, the natural gastric juice of the child, and experience confirms the magical effect.

A white tongue is not characteristic of pneumonia (I mean a clean white, like milk, distinguished from the snow white of inflammation); but your experience will call to mind many cases of this formidable disease with this anomalous attendant, and its no inconstant fellow symptoms of an acidulous action, the discharge of green bile. The chemical treatment in this case is to combine liquor potassæ or bi-tart. potass. with your other remedies.

Rheumatism has been so frequently associated with excess of acid, that theorists have, for a few years past, laid down an alkaline course of treatment—but that excess of acid in the acute, or of alkali in the chronic, is symptomatic of the disease, I utterly deny. And here, in an opinion at variance with such a name as Golding Bird, let me ask if your own observations will not join me in the assertion that there is a marked difference between rheumatism in Europe and rheumatism in Canada, particularly those of you who have had an opportunity of seeing cases in the hospitals of London as well as this country. Nothing struck me more forcibly. Not to detain you with the question just now, I may allude to the well known fact that in England the chronic form tends to rheumatic gout, while in this country it assumes the nature of palsy. However, that the excretions in some cases, and often in certain stages of the same case, will acknowledge the test of alkaline and acid excess respectively, I think I may safely state as proven; hence it is our duty to seek out the adominitions that chemistry suggests, and govern ourselves accordingly.

The powerful antiseptic and disinfecting effects of chlorine have been long known, but until the accidental discovery of chloride of pottassium, a few years ago, the different forms in which it was necessarily administered contained objections commensurate with its advantages. This salt is free from any of the difficulties of former preparations. Not so caustic for local use as chloride of lime, and more effective than the chloride of sodium, it imparts its chlorine readily, and leaves the potass. as mild a caustic and gentle stimulant as could be wished—and whenever it has been applied to fœtid and indolent ulcers, the whole array of yeast and charcoal and other carbonaceous applications have fled before it in confusion. In that modern and most dreaded disease, diphtheria, there appears to me to be no safety in any other remedy. This is a malignant fever with putrid sore throat, the whole lining surface of the fauces and pharynx throwing off a false membrane, which again immediately forms attachments in places, and thus hastens dissolution by a mechanical obstruction. Gentlemen, whose opinions I cannot but respect, still place their trust in the nitras argenti; but its application is very difficult, as it should touch *only* certain places, and its effect uncertain, while two or three free applications of the chloride of potass. with a sponge, will almost completely remove the local difficulty, and leave you a fair wind and open sea.

Thus I have viewed chemistry only as an adjunct or a chief assistant at our labors; but as we rise in the scale of disease, and find, as we do, our difficulties increase and our skill more at fault, we may be induced to look to this science as the polar star in our distress, aud the guiding spirit to carry us through the storm. To include under one general term, the different disorders of this kind, such as albuminuria, tuberculosis, phthisis, &c., I will speak alone of scrofula or general cachexia, and of course will not attempt any minutiæ of detail. We find an excess of fluid over the solid part of the body, as well as deficiency in fibrin or muscular fibre, and often total want of some important constituents of health, such as phosphorus and sulphur. Or we have excess of hydrogen, with loss of nitrogen. On the use and distribution of these two elements depend, almost solely, our hopes of cure; simply using carbonaceous and oxygenated substances as nourishment, to keep good the supply and preserve the waste, until we can affect a change in the other ingredients. That chemical changes do not take place with the same certainty and regularity of the system, influenced by vitality, as in the alembic and under our observation, I am willing to admit; but that these changes are more or less definitely and correctly effected while circulating in the blood, I think can be as clearly proven. As an instance, and as it constitutes a most important part in our curative process, give, for a few days, cod liver oil with phosphate of lime, and you will detect the dumb-bell crystals of oxalate of lime in the urine. Now, this can only be effected by the change of carbonic acid and carbonic oxide into oxalic acid, which from its stronger affinity sets free the phosphoric acid and unites with the lime. This change is easily produced in some part of the transit through the circulation.

Raw beef, pounded to shreds, has of late received the approval of the London and Continental Hospitals, as food in these cases upon physiological reasons, particularly its ready transformation with little effort of nutrition to the much needed fibrin*—but we also find that the pounding divests it of its cellular substance or cellulose, which is composed of hydrogen and oxygen in the exact proportions of warm water. So the three, carbonic, oxalic and tartaric acids, to which so much

* Mr. Hall certainly does not mean to say that fibrin and muscular fibre are one and the same thing ?—Ed.

importance has been attached, contain, two of them none, and the other a very small proportion of hydrogen, which may materially check that ready solvent from carrying the most important solids out of the system. I cannot agree with the one-man power of Dr. Churchill, about the use of hypophosphites, but have no doubt of their most important efficacy when combined with cod liver oil so as to produce the chemical transposition before mentioned.

The chemical indications of cure, therefore, consist in the proper regulations of hydrogen and nitrogen. The first by keeping from the system all such articles of diet as contain the elements of water, and using for medicines, like medical compounds, the few acids I have named. The second by conveying into the system as much as possible of substances rich in nitrogen. Of these the principal are nitric acid, nitrate and cyanide of potass., and the different preparations of ammonia, the chief of which is the muriate—with articles of diet confined to caseine of milk, albumen of egg, and fibrin from beef and mutton.

Fruit, often highly recommended, derives its principal advantage from the long mastication required, causing a greater quantity of atmospheric air to be conveyed to the stomach with the saliva.—*British Am. Jour.—Pacific Med. Jour.*

